# Cauchao Berry (*Amomyrtus luma*) as a Promising Source of Bioactive Compounds: Optimized Extraction, Phytochemical Characterization, and Assessment of Antioxidant and Antidiabetic Potential

**DOI:** 10.3390/ijms26178391

**Published:** 2025-08-29

**Authors:** Luis S. Gomez-Perez, Jacqueline Poblete, Vivian García, René L. Vidal

**Affiliations:** 1 Centro de Biología Integrativa, Facultad de Ciencias, Universidad Mayor, Santiago 8380000, Chile; 2 Escuela Nutrición y Dietética, Facultad de Medicina y Ciencias de la Salud, Universidad Mayor, Santiago 8580745, Chile; 3 Departamento de Ingeniería en Alimentos, Universidad de La Serena, La Serena 1700000, Chile; j.pobletegalleguillos@gmail.com (J.P.); vivian.garcia@userena.cl (V.G.); 4 Centro FONDAP de Gerociencia, Salud Mental y Metabolismo (GERO), Santiago 8380000, Chile

**Keywords:** Cauchao berry, optimized extraction, bio-compounds, antioxidant potential, α-glucosidase inhibition

## Abstract

The Cauchao berry (*Amomyrtus luma*), native to southern Chile and Argentina, has been traditionally used in folk medicine, yet scientific evidence supporting its bioactive potential remains limited. This study aimed to optimize the extraction of bioactive compounds and assess their antioxidant and antidiabetic properties. Fresh and freeze-dried samples were compared in terms of proximate composition, dietary fiber, reducing sugars, and fatty acid profiles. Proximate and fiber contents were determined using AOAC methods, while fatty acids were analyzed by gas chromatography, and α-tocopherol levels were measured via HPLC. Extraction optimization was conducted using a Box–Behnken design within a response surface methodology framework, employing freeze-dried samples. Total phenolic (TPC), flavonoid (TFC), and anthocyanin (TAC) contents were quantified spectrophotometrically. Antioxidant potential was assessed by DPPH and ORAC assays, while α-glucosidase inhibition determined antidiabetic activity. Phenolic profiles were characterized by HPLC. Optimal extraction conditions (58% ethanol, 60% ultrasound power, 30 min) enhanced antioxidant response. Results showed high fiber content (~39%), linoleic acid as the predominant fatty acid, and an α-tocopherol concentration of ~95 µg/g. TPC, TFC, and TAC values reached 25.43 ± 0.85, 46.51 ± 1.38, and 5.91 ± 0.40 mg/g d.m., respectively. Antioxidant capacity was 289.54 ± 9.05 μmol TE/g (DPPH) and 451.09 ± 6.04 μmol TE/g (ORAC). The IC_50_ for α-glucosidase inhibition was 0.558 ± 0.015 mg/mL. Phenolic compounds were identified. These findings position the Cauchao berry as a promising source of bioactive compounds with potential health benefits.

## 1. Introduction

Berries are widely acknowledged worldwide as some of the most nutritious foods available, thanks to their rich and diverse content of bioactive compounds. These include phenolic acids, flavonoids, and anthocyanins, which are celebrated for their numerous health-promoting properties [1]. These compounds contribute not only to the vibrant colors of berries but also to the antioxidant, anti-inflammatory, antihypertensive, and antihyperglycemic activities [2].

Chile boasts a rich variety of small fruits, particularly berries, among which the most notable are murta, blueberries, maqui, and calafate, among others. Researchers have increasingly focused on these berries because of their exceptional functional properties, including strong antioxidant activity, anti-inflammatory benefits, and potential positive effects on cardiovascular and metabolic health [1]. Their distinctive composition, abundant in bioactive substances such as polyphenols, vitamins, and dietary fiber, has captured the interest of scientists and healthcare experts globally, making them valuable candidates for use in functional foods and nutraceutical innovations.

However, other wild berries, such as Cauchao berries (*Amomyrtus luma*), remain largely unexplored, presenting a promising opportunity for further scientific research. These fruits could emerge as a new source of bioactive compounds with potential functional properties that may contribute to improved health and wellness. The Cauchao or Chauchao berry is a native and endemic species of southern Chile and Argentina. It thrives in rainforests, particularly near streams and in other high-altitude, humid areas. This globose berry has a pleasant flavor and aroma, transitioning from reddish to blackish as it ripens. Currently, the fruit is primarily consumed fresh or as part of artisanal products, including jams, desserts, juices, pastries, and fermented alcoholic beverages. Historically, it has been used by indigenous peoples for its stimulating and astringent properties [3]. Moreover, studies from ancestral Mapuche medicine report that its leaves are used to lower blood pressure and cholesterol levels and to treat liver diseases [4]. The leaf extract has also been found to contain aromatic compounds such as linalool, 1,8-cineole, and β-caryophyllene, among others [5]. These compounds, widely recognized for their antioxidant, anti-inflammatory, and antimicrobial activities, provide a biochemical rationale for considering *Amomyrtus luma* as a potential source of functional agents. In this context, the fruit, although still underexplored, may also contain bioactive molecules with health-promoting effects, reinforcing the need for scientific investigation into its composition and properties. To date, no peer-reviewed studies in high-impact journals have analyzed the bioactive profile or functional properties of *Amomyrtus luma* fruit.

On the other hand, environmentally friendly extraction methods have been employed to obtain new antioxidant concentrates or natural colorants from berries [6,7]. Technologies such as ultrasound have been explored to reduce extraction time and energy consumption while avoiding the use of high temperatures [8]. Ultrasound-assisted extraction utilizes acoustic cavitation, and when applied at low frequencies (20–100 kHz), it generates large bubbles that implode violently, leading to enhanced cell disruption, improved solvent penetration, and consequently higher extraction yields [9]. This technique is considered an effective alternative for extracting phenolic compounds and anthocyanins from maqui berries [10], as well as from aronia and grapes [11]. Therefore, ultrasound-assisted extraction presents a promising method for obtaining Cauchao berry extracts. Nonetheless, the extraction conditions must be carefully optimized to maximize bio-compound yield.

Berry consumption is currently on the rise, driven by their rich content of bioactive compounds that offer various health benefits for humans. These benefits include antioxidant, anti-inflammatory, and cardioprotective properties, which contribute to reducing the risk of chronic diseases such as cardiovascular conditions, diabetes, and certain types of cancer [12]. However, these properties have been scarcely explored in Cauchao berries. The present study evaluated the antioxidant and antidiabetic potential of extracts rich in bioactive compounds derived from Cauchao berries by investigating these properties. This research aims to provide valuable insights into the health-promoting capabilities of this underexplored fruit.

## 2. Results

### 2.1. Proximate Composition and Reducing Sugars

The proximate analysis, shown in Table 1, was conducted on fresh and freeze-dried Cauchao berry samples. The fresh sample exhibited a moisture content comparable to other berries, such as murtilla [13], but higher than that of blueberries and maqui, which have a moisture content of approximately 65 g/100 g on a wet basis [14,15]. The content of ash, crude protein, carbohydrates, crude fiber, and fats was significantly more concentrated in the freeze-dried sample due to the reduced water content in the matrix.

As expected, a high concentration of sugars was observed in the freeze-dried Cauchao berries. The fresh value was significantly higher compared to other fruits, such as strawberries, blackberries, raspberries, and cranberries, while being very similar to that of blackcurrants [16].

### 2.2. Dietary Fiber Content

Dietary fiber refers to the edible components of plants that resist digestion and absorption in the human small intestine. Instead, these fibers remain intact as they pass into the large intestine, where they may undergo complete or partial fermentation [17]. The contents of soluble (SDF), insoluble (IDF), and total (TDF) dietary fiber were determined by the AOAC method. Soluble dietary fiber, which includes components such as non-cellulosic polysaccharides, oligosaccharides, pectins, β-glucans, and gums, is highly valued for its nutritional benefits. IDF includes cellulose, parts of hemicellulose, and lignin [18]. Table 1 shows the SDF, IDF, and TDF for fresh and freeze-dried Cauchao berries. As expected, no differences were observed between the evaluated samples, as both underwent dehydration according to the prescribed methodology. Notably, the TDF value was considerably higher than that reported for comparable fruits, such as murta berries. For instance, murta berry has a TDF value of 17.55 ± 0.37 g/100 g dry matter (d.m.) based on the same determination method [19]. In comparison, the Cauchao berry demonstrated approximately twice the total dietary fiber, highlighting its superior nutritional profile and potential as a rich dietary fiber source.

### 2.3. Fatty Acids Profile and α-Tocopherol Content

Vegetable fatty acids, which are part of the essential unsaturated fatty acid group, show considerable biological activity. These fatty acids are predominantly found in seeds and oils extracted from fruits such as berries and others [20]. These essential nutrients play a critical role in various bodily functions, including maintaining healthy cell membranes, supporting cardiovascular health, and regulating inflammation [21]. Since the human body cannot synthesize these fatty acids on its own, consuming them through a balanced diet is vital for overall well-being. Table 2 shows the fatty acid profile of the Cauchao berry. Six fatty acids were identified, and no differences were observed between fresh and freeze-dried berries. The profile found is very similar to those reported in murta berries in terms of fatty acids and their quantities [18]. It stands out for its linoleic acid (LA, C18:2n6c) content, which is approximately 80%. LA, an essential fatty acid that must be obtained through the diet [22], is the primary polyunsaturated fatty acid found in Cauchao berries. Oleic acid was found in the second proportion (OA, C18:1n9c), the quantity found was higher than other similar berries such as blueberries, red gooseberries and black currants [23]. The third portion identified was palmitic acid (PA, C16:0), which is one of the most common saturated fatty acids in nature, found in animal and human tissues, as well as in plants, algae, fungi, yeasts, and bacteria [24]. Essential plant-derived fatty acids, including linoleic acid, oleic acid, and palmitic acid, play pivotal roles in human health. Consuming Cauchao berries, which are rich in these fatty acids, may confer substantial health benefits.

α-Tocofpherol is one of the primary components of vitamin E, essential for meeting human nutritional requirements, and is primarily obtained through food [25]. Table 2 presents the α-tocopherol content of both fresh and freeze-dried Cauchao berries, with the latter showing a slightly higher concentration, likely due to the concentration effect of solutes caused by the drying process. The α-tocopherol concentration in both forms exceeds that reported in other similar fruits, such as murta berry with values between 1.06 ± 0.02 and 5.80 ± 0.15 mg/100 g [26] and maqui berry with values between 17.1  ±  0.8 μg/g and 31.7  ±  0.5 μg/g [27].

### 2.4. RSM Method

The response surface modeling method was used to determine the optimal condition of the antioxidant capacity of the Cauchao berry extract. Table 3 presents the factors and levels used in the Box–Behnken model, showing their impact on the antioxidant capacity of Cauchao berry extract, measured by the DPPH method. The results indicated that antioxidant potential varies based on these factors, emphasizing the need to optimize extraction parameters such as time, ultrasonic power, and solvent concentration to maximize antioxidant compound yield and antioxidant capacity.

**Figure 1:**
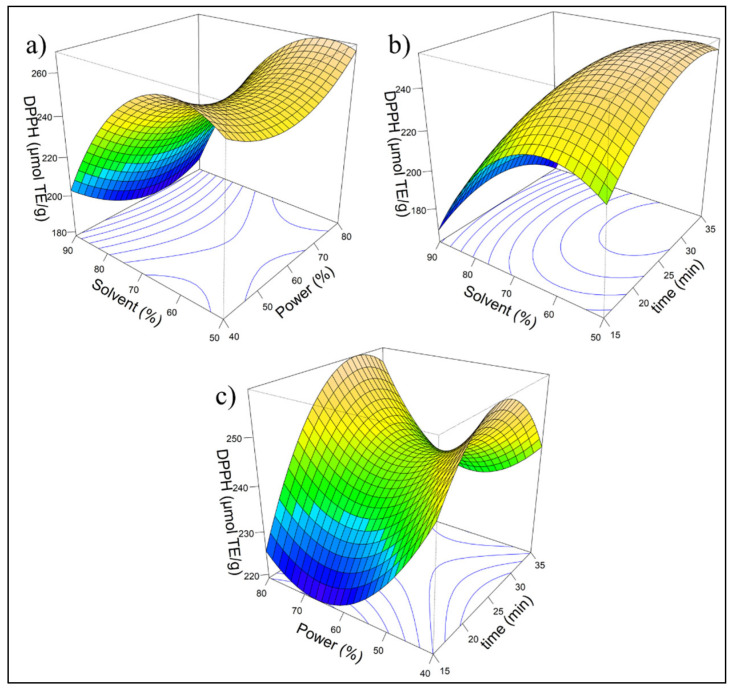
Response surface for antioxidant capacity (DPPH): (**a**) Effect of solvent (%) and power (%) on DPPH; (**b**) effect of solvent (%) and time (min) on DPPH; (**c**) effect of power (%) and time (min) on DPPH. The color gradient, ranging from blue to brown, with blue representing lower DPPH values and brown representing higher values.The response surface model for antioxidant capacity measured by the DPPH assay is described by the following quadratic equation:.

Figure 1 shows three response surface plots illustrating the impact of the independent variables: solvent (%), power (%), and time (min), on the response variable, DPPH (μmol TE/g). Each plot represents the connection between two independent variables while keeping the third constant, enabling the visualization of individual effects and interactions among the evaluated factors. The color gradient, ranging from blue to brown, indicates response variability, with blue representing lower DPPH values and brown representing higher values.

The model fitted using RSM indicates that the linear term of solvent (X_1_) was highly significant (*p* < 0.001), demonstrating its strong influence on the response, whereas power (X_2_) and time (X_3_) did not show significant effects in the linear model. However, the quadratic terms of X_1_, X_2_, and X_3_ are significant, suggesting a nonlinear relationship with an optimal point. The adjusted R^2^ was 0.7855, indicating a good explanatory power, although additional factors may contribute to variability. ANOVA confirms that the first-order effects are significant (*p* = 0.00264), while interaction effects are not, ruling out synergistic influences. The quadratic effects are significant (*p* = 0.01292), demonstrating a curvilinear response pattern. The stationary point suggested that maximum antioxidant activity was achieved with 57.58% solvent, 55.96% power, and 29.29 min of extraction, underscoring the importance of these parameters for process optimization. To enhance laboratory reproducibility, the parameters were adjusted to 58% solvent, 60% power, and 30 min of extraction.

(1) $$DPPH=242.55-33.71x_{1}-1.94x_{2}+7.36x_{3}-7.16x_{1}x_{2}-10.59x_{1}x_{3}\;+7.82x_{2}x_{3}-29.63x_{1}^{2}+14.51x_{2}^{2}-14.40x_{3}^{2}\;$$

**Figure 2:**
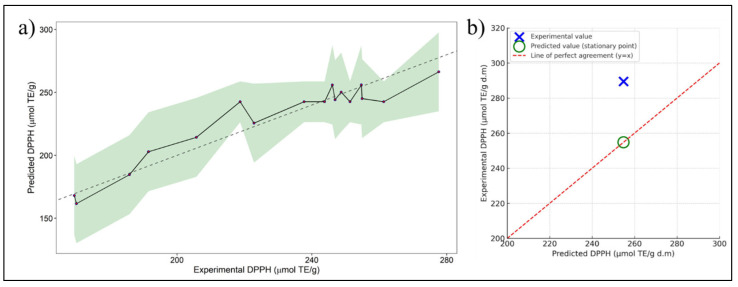
(**a**) Experimental against predicted values of DPPH. The solid black line connects the predicted DPPH values, while the green shaded area represents the confidence interval (uncertainty range) of the prediction. The dashed gray line indicates the line of perfect agreement (*y = x*) for reference. (**b**) Model validation graph comparing predicted and experimental DPPH values at optimal conditions. The large blue cross indicates the experimental value, while the green circle shows the predicted value (stationary point). The red dashed line represents the ideal 1:1 correlation (*y = x*), used to visually assess model accuracy.

This model accounts for linear, quadratic, and interaction effects, providing a mathematical representation of the relationship between the experimental factors and the DPPH response. Figure 2a depicts the model fit by comparing the original experimental DPPH values against the predicted ones (dashed black line), including their range of variability (green shading).

### 2.5. Total Content of Phenolics, Flavonoids, and Anthocyanin

The total bioactive compounds present in the Cauchao berry extract, including total phenolic content, total flavonoid content, and total anthocyanin content, were determined in the extract obtained under optimized conditions according to the RSM results. Table 4 shows the TPC, TFC, and TAC results. Phenolic compounds present in plant-based foods are classified as flavonoids, stilbenes, lignans, phenolic acids, and others, distinguished by their chemical structures [28]. The TPC value of the Cauchao berry extract was 25.43 ± 0.85 mg GAE/g d.m., equivalent to 2543 mg GAE/100 g d.m. This value is considerably higher than those reported for fresh fruits such as strawberry (317–443 mg/100 g), blackberry (411–678 mg/100 g), blueberry (154–585 mg/100 g), blackcurrant (817–1042 mg/100 g) and raspberry (192–529 mg/100 g) [16].

The TFC of the optimized Cauchao berry extract was 46.51 ± 1.38 mg QE/g d.m., indicating a substantial concentration of these secondary metabolites. When compared to other similar native Chilean berries, such as maqui (*Aristotelia chilensis*) and murta (*Ugni molinae*), which exhibit TFC values ranging from 43.06 ± 0.54 to 55.56 ± 5.60 mg QE/g d.m., respectively [19,29], the Cauchao berry demonstrated comparable levels. The TFC value highlights the potential of Cauchao berries as a competitive source of flavonoids among native berry species.

The TAC value was 5.91 ± 0.40 mg cyanidin-3-glucoside/g d.m. When compared to other berries, such as murta (6.85 mg cya-3-glu eq./g) [1], blackberry (2.10 mg cya-3-glu eq./g), blackcurrant (7.59 mg cya-3-glu eq./g), and different blueberry cultivars (values between 0.03 and 0.66 mg cya-3-glu eq./g) [23]. Cauchao berry presents TAC values higher than most and comparable to those of murta and blackcurrant. This anthocyanin content supports its potential as a promising natural source of compounds beneficial to human health.

### 2.6. Antioxidant Potential

Table 4 shows the antioxidant potential of optimized Cauchao berry extract evaluated by DPPH and ORAC assays. The antioxidant potential of the Cauchao berry extract was remarkable, with values of 289.54 ± 9.05 μmol TE/g d.m. and 451.09 ± 6.04 μmol TE/g d.m. for DPPH and ORAC, respectively. When compared to another similar berry, such as murta (DPPH: 220 μmol TE/g d.m.; ORAC: 599.5 μmol TE/g d.m.) [29,30], blueberry (DPPH: 125.79 μmol TE/g d.m.; ORAC: 443.01 μmol TE/g d.m.) [31], fresh blackberry (DPPH: 295.8 ± 1.9 μmol TE/g; ORAC: 63.7 ± 0.2 μmol TE/g), and dehydrated blackberry (DPPH: 1203.8 ± 27.9 μmol TE/g d.m.; ORAC: 236.8 ± 1.0 μmol TE/g d.m.) [32], the Cauchao berry presents equivalent values.

### 2.7. α-Glucosidase Activity

**Figure 3:**
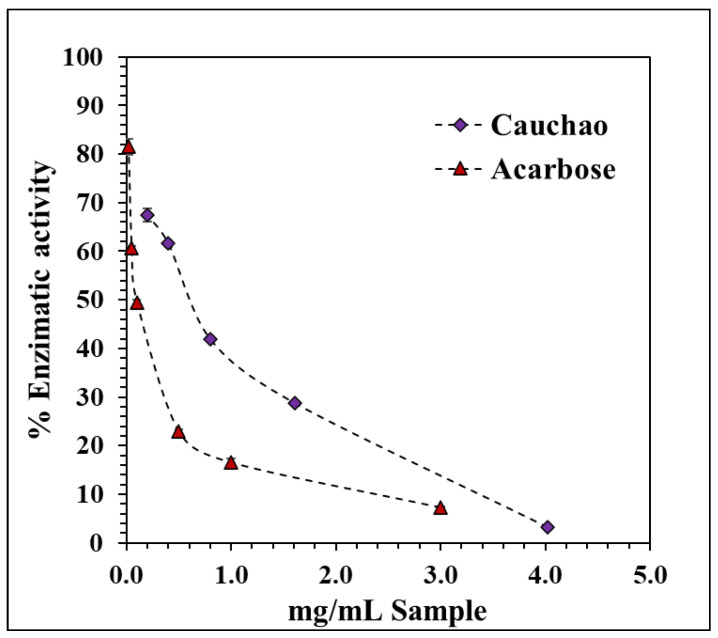
Inhibitory effect of Cauchao berry extract on α-glucosidase activity. Values are averages (*n* = 3), and error bars are standard deviation.

The enzyme α-glucosidase plays a key role in the hydrolysis of complex carbohydrates into simpler, absorbable sugars. Inhibiting its activity is an effective strategy to delay intestinal glucose absorption, thereby reducing postprandial glycemic spikes and potentially slowing the progression of type 2 diabetes mellitus [33]. The Cauchao extract demonstrated a clear, concentration-dependent inhibition of α-glucosidase activity, with a progressive decline in enzymatic function from 67% to nearly 5% at concentrations of 4 mg/mL (Figure 3). The resulting inhibition curve exhibited a decreasing hyperbolic kinetic profile, fitted using a nonlinear exponential model, yielding an IC_50_ of 0.558 ± 0.015 mg/mL, indicative of high inhibitory potency at relatively low concentrations. This value is competitive with other berry extracts reported in the literature, including mulberry fruit (IC_50_ = 0.62 ± 0.06 mg/mL) [34], fermented murta berry juice (IC_50_ = 805 ± 20 µg/mL) [35], and freeze-dried maqui extract (IC_50_ = 0.315 ± 0.046 mg/mL) [27]. Notably, the strong inhibitory effect of the Cauchao extract was observed despite it being a crude, non-fractionated preparation. Although its potency is significantly lower than that of acarbose (IC_50_ = 0.057 ± 0.005 mg/mL), a synthetic reference inhibitor.

### 2.8. Phenolic Compound Profiles

**Figure 4:**
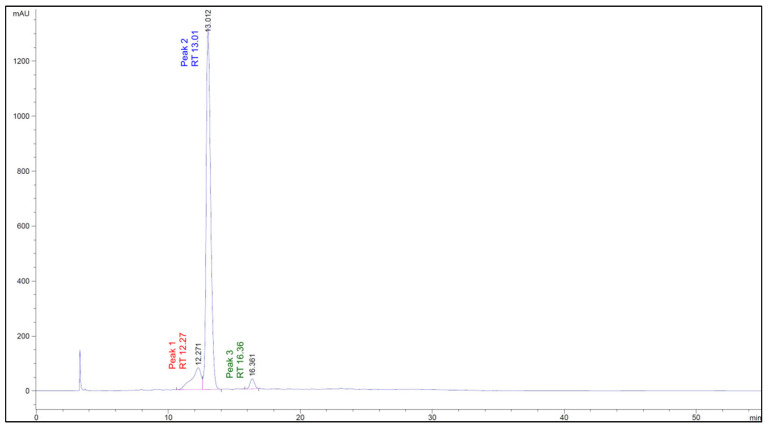
Chromatographic profile of the Cauchao berry extract at 520 nm, where three major peaks were detected at retention times of 12.27, 13.01, and 16.36 min, respectively.

The chromatographic profile of the Cauchao extract at 520 nm (Figure 4) revealed three well-defined peaks at retention times of 12.27, 13.01, and 16.36 min, respectively. The major peak, eluting at 13.01 min and representing over 85% of the total area, was tentatively identified as chlorogenic acid, a hydroxycinnamic acid derivative, with a mean concentration of 3.17 mM. The smaller peaks at 12.27 and 16.36 min were associated with cyanidin, a representative anthocyanin (0.026 mM), and rutin, a flavonol glycoside (0.049 mM), respectively. A fourth compound, sinapic acid (a hydroxycinnamic acid), was also quantified at a relatively high mean concentration (1.52 mM), yet was not clearly visualized in the 520 nm chromatogram. This discrepancy is likely due to its distinct spectral behavior, as hydroxycinnamic acids, such as sinapic acid, exhibit maximal absorbance in the 238–326 nm range [36] and, therefore, are poorly detected at anthocyanin-specific wavelengths. Altogether, these results highlight the co-occurrence of representative phenolic and anthocyanin compounds in the Cauchao extract, supporting its potential as a source of multi-functional bioactive compounds.

## 3. Discussion

This discussion interprets the results in light of current scientific evidence, highlighting the physiological, functional, and technological relevance of Cauchao berries. The findings are contextualized through biochemical, nutritional, and health-related perspectives, with comparisons to similar fruit matrices.

### 3.1. Proximal Composition, Reduces Sugar and Dietary Fiber

The proximal content shows that the freeze-dried Cauchao exhibited a significantly lower moisture content, which is essential for extending the fruit’s shelf-life by reducing microbial activity and slowing enzymatic processes. This characteristic highlights freeze-drying as an effective preservation method, particularly for retaining the berries’ quality and nutritional value over time. The levels of crude fiber and fats are particularly noteworthy, as they suggest a high dietary fiber content and the presence of relevant fatty acids, respectively. These components are crucial due to their potential health benefits, including promoting digestive health and enhancing the overall nutritional profile. On the other hand, Cauchao berries are a rich source of sugars compared to other fruits with similar characteristics. Sugars are vital to plants, serving both as essential nutrients and as central signaling molecules that regulate gene expression involved in growth, development, metabolism, stress responses, and disease resistance [37].

In terms of dietary fiber, the IDF content was found to be significantly higher than that of SDF. This distinction is particularly noteworthy, as IDF has been widely recognized for its beneficial effects on human health. Studies have linked its consumption to a reduced risk of developing various diseases, including cardiovascular conditions and type 2 diabetes [38]. Additionally, IDF plays a critical role in promoting digestive health by improving bowel regularity, preventing constipation, and improving the production of short-chain fatty acids [39]. On the other hand, soluble dietary fiber is readily available for fermentation by fiber-degrading microorganisms in the intestine. This process results in the production of a variety of beneficial metabolites, such as short-chain fatty acids (SCFAs), which play a crucial role in maintaining gut health and other physiological functions such as increased satiety and reduced energy intake, which leads to control appetite, improve insulin sensitivity, and reduce weight [40]. Also, soluble dietary fibers can bind to metal ions like calcium, magnesium, iron, copper, and zinc, transporting them to the distal colon. As SDFs are degraded by local bacteria, the released ions help inhibit pathogens, enhance gut microbiota diversity, and protect against infections [40]. According to the mentioned fiber properties, Cauchao berries could offer significant benefits that support gut health.

### 3.2. Fatty Acids Profile and α-Tocopherol Content

The main fatty acids identified in Cauchao berry include linoleic acid, oleic acid, and palmitic acid. These fatty acids are important for human health. Recent studies have suggested that LA may exhibit neuroprotective effects in both in vitro and in vivo models of Parkinson’s disease, potentially by modulating lipid droplet dynamics [41]. On the other hand, it has also been shown that the consumption of LA reduces the risk of cardiovascular diseases in healthy individuals [42]. In the brain, OA is a key component of membrane phospholipids and is abundantly present in myelin. A notable reduction in OA levels has been observed in the brains of individuals suffering from major depressive disorders and Alzheimer’s disease [43]. This decrease in OA may contribute to the pathophysiology of these conditions, as it plays a crucial role in maintaining neuronal function and integrity. Additionally, it has been suggested that OA may help lower blood pressure by enhancing endothelial function and may also reduce the risk of developing rheumatoid arthritis. Oleic acid is well-known for its health benefits, including its ability to regulate blood glucose levels, lower blood pressure, and provide anti-inflammatory and antimicrobial effects [44]. Palmitic acid is a crucial component of triglycerides in adipose tissue, and its presence in blood carries significant diagnostic implications. High consumption of palmitic acid has been associated with an increased risk of cardiovascular disease (CVD) and elevated levels of LDL and HDL cholesterol. Studies indicate that its intake correlates with a higher risk of heart failure and mortality due to CVD. PA promotes inflammation, contributing to the progression of CVD and cardiac damage [45]. However, recent studies have reported that palmitic acid (PA) has the potential to inhibit the proliferation of cancer cells, including those from prostate, gastric, and breast cancers, suggesting its potential as a basis for developing new therapeutic options for the treatment of various cancer types [46]. Linoleic acid, present in high concentrations in these berries, is not only essential for cardiovascular health but may also exhibit neuroprotective effects. Additionally, oleic acid, known for its antioxidant and anti-inflammatory properties, contributes to the regulation of blood pressure and supports brain health. While excessive consumption of palmitic acid is associated with certain health risks, it is present in relatively low amounts in these berries and has been noted for its potential anti-tumor properties. Including these berries in the diet could represent a beneficial strategy for enhancing overall well-being, mitigating the risk of cardiovascular diseases, and supporting neuronal function.

α-Tocopherol is widely recognized for its antioxidant properties, as it neutralizes reactive oxygen species and prevents lipid peroxidation, thereby protecting cell membranes from oxidative damage [47]. This antioxidant action is essential for maintaining cellular integrity. Moreover, α-tocopherol may help prevent cardiovascular disease by modulating signaling pathways and reducing cell proliferation and oxidative stress. It also inhibits the uptake of oxidized low-density lipoprotein (oxLDL) by immune cells, preventing foam cell formation. Additionally, it decreases inflammation and apoptosis in macrophages. However, some studies suggest that it does not reduce lipid peroxidation in cardiovascular lesions [48]. In terms of its neuroprotective effects, α-tocopherol may contribute to the prevention and management of Alzheimer’s disease (AD). Studies have shown that α-tocopherol reduces β-amyloid formation, exhibits antioxidant and anti-inflammatory properties, and supports mitochondrial function, potentially slowing AD progression. Additionally, higher intake has been linked to a reduced risk of dementia and mild cognitive impairment [49]. Given its significant antioxidant, cardiovascular, and neuroprotective benefits, α-tocopherol stands out as a vital bioactive compound with potential therapeutic applications. The high concentration found in Cauchao berries, particularly in their freeze-dried form, underscores their nutritional value as a natural source of vitamin E.

### 3.3. RSM Method

In the optimization analysis of the extraction process of bio-compounds with RSM; a stationary point was identified at X_1_ = −0.6209, X_2_ = −0.2019, and X_3_ = 0.4289 which, based on eigenanalysis, was characterized as a saddle point. These findings suggest that the response does not exhibit a clear absolute maximum within the range of the factors. However, this stationary point represents the most favorable predicted response within the studied conditions. The experimental value of DPPH at optimal conditions was 289.54 μmol TE/g d.m. (Table 4) and was higher than the value predicted by the model (254.79 μmol TE/g d.m.), as shown in Figure 2b, with an absolute error of 13.64%. This difference can be attributed to natural experimental variability or to the presence of factors not fully accounted for by the quadratic model, such as variations in raw material composition. Nevertheless, the model proved to be a useful predictive tool, pointing toward experimental conditions that led to an even greater antioxidant response. These results confirm the practical applicability of the model for process optimization.

### 3.4. Total Content of Phenolics, Flavonoids, and Anthocyanin

The high concentration of TPC observed in the Cauchao berry extract could be due to the freeze-drying process, which eliminates water content and concentrates bioactive compounds, as well as to the optimization of extraction conditions, which allowed greater recovery of phenolic compounds. These bioactive compounds are found in various dietary sources, such as tea, fruits, and vegetables, and have been shown to offer potential health benefits. Their antioxidant properties contribute to glycemic control, blood pressure regulation, and improved lipid profiles [50]. Additionally, phenolic compounds, due to their nontoxic nature, play a key role in neuroprotection, acting through antioxidant, anti-inflammatory, and autophagy-modulating mechanisms. In animal models, they have demonstrated the ability to inhibit biomarkers of inflammation, oxidative stress, and neurotoxicity. Furthermore, they regulate various signaling pathways involved in immune and neuronal responses. Their effects include protecting neurons from neurotoxins, reducing neuroinflammation, and improving memory and cognitive function [51]. The TPC value not only positions Cauchao berry as a natural source rich in phenolic compounds but also suggests its possible application in the prevention of diseases associated with oxidative stress and neurodegenerative disorders. Flavonoids constitute a class of polyphenolic compounds widely distributed in the cells and surfaces of various plant tissues. Beyond their role in plant pigmentation, these compounds are distinguished by their importance as bioactive agents involved in multiple physiological functions [52]. Flavonoids exhibit diverse biological activities, including antioxidant, anti-inflammatory, antimicrobial, cardioprotective, and anticancer effects, largely attributed to their hydroxyl groups. Compounds such as quercetin, myricetin, and apigenin contribute to the reduction of oxidative stress, cholesterol, and the risk of cardiovascular and chronic diseases [53]. Some flavonoids are being studied for their neuroprotective properties, associated with their antioxidant and anti-inflammatory properties. These compounds can cross the blood-brain barrier and reach specific brain regions. Their therapeutic potential is highlighted in neurodegenerative and ischemic diseases, such as Alzheimer’s, Parkinson’s, and stroke [54]. Several natural flavonoids have demonstrated neuroprotective effects by reducing oxidative damage, regulating mitochondrial dysfunction, and modulating cellular pathways, such as Nrf2 (nuclear factor erythroid-related factor 2), ERK (extracellular signal-regulated kinase), and Akt (protein kinase B). Compounds such as catechins, ampelopsin, and pinocembrin stand out for their ability to prevent neuronal death. Therefore, flavonoids are emerging as promising therapeutic agents against neurodegeneration in Parkinson’s disease [55]. Anthocyanins are natural pigments that give fruits, leaves, and flowers their characteristic red, blue, and purple colors. In addition, they are notable for their potent antioxidant and anti-inflammatory effects, helping to prevent diseases such as diabetes, obesity, hypertension, and certain types of cancer, especially colon cancer. These benefits are largely due to their ability to block NF-κB (nuclear factor kappa-light-chain-enhancer of activated B cells) mediated inflammatory pathways, thus helping to maintain cardiovascular and metabolic health. However, their effect may vary depending on the specific structure of each compound [56]. Anthocyanins are also notable for their potential to combat neurodegenerative diseases. These pathologies share processes such as brain inflammation, oxidative stress, and neuronal damage due to excessive stimuli, where anthocyanins act by blocking and modulating these pathways. Therefore, they are emerging as promising allies in protecting and improving neurological health [57]. The remarkable anthocyanin content of the Cauchao berry, comparable to that of other recognized fruits, supports its potential as a natural source of bioactive compounds with health benefits. These results not only confirm the bioactive richness of the Cauchao berry, but also open a promising field of research towards its nutraceutical and neuroprotective use in humans.

### 3.5. Antioxidant Potential

Antioxidant potential comes from key compounds (phenols and flavonoids present in fruits and vegetables) that prevent the oxidation of lipids, proteins, and other biomolecules, protecting both food and the human body from damage caused by reactive oxygen species (ROS). In foods, they extend shelf-life while maintaining flavor, color, and texture. In humans, they reduce the risk of chronic diseases such as cardiovascular disease. Antioxidant efficacy depends on their chemical structure, bioaccessibility, and metabolism [58]. It is important to consider that the evaluation of antioxidant potential using the DPPH and ORAC methods provide complementary information, as both are based on different action mechanisms and, therefore, cannot be compared. The determination of the antioxidant capacity of natural extracts can vary considerably depending on the in vitro method used. This variability is due to the fact that no single assay is capable of considering the complexity of the antioxidant mechanisms involved, the distribution of compounds in heterogeneous matrices, or the influence of other components present in the system. Therefore, the use of more than one complementary analytical method is recommended to obtain a more robust and objective assessment of antioxidant capacity [59].

The DPPH assay measures the capacity of antioxidants to donate electrons or hydrogens to a free radical, reflected by the decrease in absorbance assessed at 515–520 nm. The DPPH radical does not exist in biological systems and can interfere, especially in the presence of certain solvents, which can underestimate antioxidant activity. However, it is simple, inexpensive, rapid, and widely used to compare the antioxidant activity of extracts or pure compounds. On the other hand, the ORAC method evaluates the ability of compounds to neutralize peroxyl radicals generated by AAPH (2,2′-azobis(2-methylpropionamidine) dihydrochloride) through the oxidative degradation of a fluorescent molecule (such as fluorescein). The loss of fluorescence indicates oxidative damage, while the presence of antioxidants reduces this loss [60]. The comparable values obtained in similar fruits in both assays indicate that the Cauchao berry possesses a combination of compounds capable of acting through different mechanisms, which increases its relevance from a nutritional and functional perspective. In this context, the results obtained reinforce the potential application of this berry as a bioactive ingredient in the development of functional foods or nutraceuticals aimed at preventing cellular oxidative damage, with a potentially positive impact on human health.

### 3.6. α-Glucosidase Activity

A notable inhibitory effect of Cauchao berry extract on the enzyme α-glucosidase was observed. Although the precise mechanism of inhibition is not clarified in this study, this effect may be attributed to synergistic interactions among multiple phenolic constituents in the Cauchao berry matrix, which is consistent with the complex phytochemical profile of native berries. From a functional standpoint, this type of enzyme inhibition is particularly relevant for postprandial glycemic regulation, as early modulation of carbohydrate digestion can help mitigate glucose surges following meals. Accordingly, Cauchao extract emerges as a promising candidate for integration into functional foods or nutraceutical products targeting dietary glycemic control, particularly in individuals at risk of insulin resistance or type 2 diabetes. Although its inhibitory potency is lower than that of acarbose (IC_50_ = 0.057 ± 0.005 mg/mL), a clinically used α-glucosidase inhibitor, the Cauchao extract demonstrated stronger activity than several other natural berry extracts reported in the literature, highlighting its competitive potential despite being a crude, unfractionated preparation.

Furthermore, a strong association has been reported between metabolic regulation and the progression of neurodegenerative diseases, including Parkinson’s disease. It has been noted that type 2 diabetes not only increases the risk of developing Parkinson’s disease but also accelerates its clinical progression [61]. Given that this condition is amenable to dietary and pharmacological interventions, the inhibitory effect of Cauchao extract on the α-glucosidase enzyme is particularly relevant for future studies exploring potential neuroprotective effects.

### 3.7. Phenolics Compound Profile

In this study, the phenolic profile of Cauchao was analyzed using high-performance liquid chromatography. The results revealed the presence of several bioactive phenolic compounds, including chlorogenic acid, sinapic acid, cyanidin and its glycosylated derivatives, and rutin. Chlorogenic acid (CGA) was the main component identified in the Cauchao extract. This compound, an ester of caffeic acid and quinic acid, is one of the most abundant phenolic acids in nature and is found in foods such as coffee, eggplant, tomato, and various fruits [62]. CGA has been widely studied for its antioxidant and anti-inflammatory properties. CGA has been shown to modulate the production of inflammatory mediators such as TNF-α, IL-1β, IL-6, IL-8, NO, and PGE2, and regulate key signaling pathways such as NF-κB, MAPK, and Nrf2, providing protective effects against cardiovascular disease and diabetes mellitus [63]. Furthermore, CGA has been reported to exert neuroprotective effects in zebrafish (MPTP) and SH-SY5Y (6-OHDA) cell-based Parkinson’s disease models, attenuating the loss of dopaminergic neurons, decreasing apoptosis, and improving locomotion [64]. Likewise, kinetic studies have shown that CGA significantly inhibits β-amyloid (Aβ) aggregation, which is implicated in the pathophysiology of Alzheimer’s [65]. This evidence supports the potential of Cauchao extract as a source of bioactive compounds, particularly in the prevention and management of diseases related to oxidative stress and inflammation.

Sinapic acid (SA) was the second major compound identified in the Cauchao extract. This compound is a hydroxycinnamic acid and is present in various plant sources, including fruits such as lemons, oranges, and berries, and is also present in cereals and oilseeds [66]. This compound has been widely studied for its antioxidant, anti-inflammatory, and neuroprotective properties. SA has been reported to block LPS-induced activation of mitogen-activated protein kinases (MAPKs) and protein kinase B (AKT) signaling [67]. Furthermore, it has been shown to exert neuroprotective effects in human neuroblastoma (SHSY5Y), with mitigation of anti-inflammatory markers such as IL-1β and TNF-α and decreased ROS levels [68]. The identification of chlorogenic acid and sinapic acid as the major compounds in the Cauchao extract provides preliminary information on its functional potential. While these findings do not allow direct biological effects to be established, they do suggest that the extract could constitute an interesting natural source of bioactive compounds relevant to health.

Finally, the freeze-dried Cauchao berry extract demonstrates a rich and diverse profile of bioactive compounds, including phenolics, flavonoids, anthocyanins, and essential fatty acids, which collectively contribute to its remarkable antioxidant and metabolic properties. The evidence presented highlights not only the berry’s nutritional value but also its promising applicability in the development of functional foods, nutraceuticals, and preventive strategies for oxidative stress-related diseases. Future studies are warranted to further explore the bioavailability, molecular mechanisms, and clinical efficacy of Cauchao-derived compounds, paving the way for their potential integration into therapeutic and dietary interventions aimed at improving human health.

## 4. Materials and Methods

### 4.1. Raw Material

**Figure 5:**
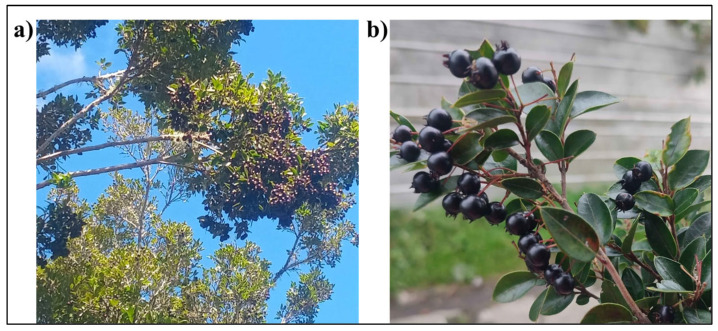
*Amomyrtus luma* (Cauchao berries). (**a**) General view of the *Amomyrtus luma* tree bearing ripe fruits, photographed in its natural environment in southern Chile. (**b**) Close-up of ripened Cauchao berries on the plant. Author: Marisol Runiahue. Date: February 2025.

The fruits of *Amomyrtus luma* (Cauchao berry), as shown in Figure 5, were obtained from artisanal gatherers located in the rural area of Chiloé Island in southern Chile. These gatherers harvest the fruit directly from wild trees that grow naturally on their ancestral lands. The species is well known and traditionally used by local communities, as documented by Fredes et al. (2024) [69], who describe the cultural significance and ethnobotanical recognition of *A. luma* among women gatherers in the extreme south forests of Chile. The plant was identified based on morphological features and traditional knowledge consistent with the literature. The berries were cleaned and carefully selected to ensure they were free from physical damage. One portion was stored in plastic bags and refrigerated at 4 °C until use, while another portion was freeze-dried and stored in a sealed plastic bag at −18 °C until needed.

### 4.2. Proximate Composition and Reducing Sugars

Lipid content was measured through gravimetric analysis after Soxhlet extraction. Crude fiber was determined by performing acid and alkaline hydrolysis on insoluble residues. The crude protein content was assessed using the Kjeldahl method, applying a conversion factor of 6.25. Crude ash content was quantified by incinerating the sample in a muffle furnace at 550 °C. Moisture content was analyzed gravimetrically. All procedures adhered to the guidelines set by the Association of Official Analytical Chemists [70]. Available carbohydrates were calculated by subtracting other components, and all analyses were conducted in triplicate. On the other hand, the reduced sugar content was determined using the Miller method [71], which involves the use of dinitrosalicylic acid (DNS) reagent and spectrophotometric measurement at 540 nm. The analysis was performed in triplicate.

### 4.3. Dietary Fiber Content

The determination of soluble dietary fiber (SDF) and insoluble dietary fiber (IDF) was conducted using the gravimetric-enzymatic method described in AOAC method No. 991.43. The analysis employed a Total Dietary Fiber Test Kit (TDF100A, Sigma-Aldrich, St. Louis, MO, USA) in conjunction with an Enzymatic Digestion Unit and Filtration System (VELP Scientifica, GDE-CSF6, Usmate, Italy). Total dietary fiber (TDF) was calculated as the sum of SDF and IDF. All analyses were performed in triplicate to ensure accuracy.

### 4.4. Fatty Acids Profile and α-Tocopherols Content

The oil sample from maqui was extracted following the Bligh and Dyer method (1959) [72]. Fatty acid methyl esters were prepared by saponifying the oil sample with 2 N KOH in methanol, followed by vortex agitation for 1 min. Methylated fatty acids were then extracted using 300 µL of n-hexane with an additional agitation step lasting 3 min. The resulting supernatant was analyzed using a gas chromatograph (Shimatzu, GC-2010 Plus, Kyoto, Japan) equipped with a flame-ionization detector. The chromatographic conditions for the analysis were as follows: an SP 2560 column was used with a detector temperature set at 260 °C and an injector temperature at 250 °C. The oven temperature program began at 100 °C, held for 5 min, followed by an increase of 4 °C/min until reaching 240 °C, which was maintained for 30 min. Helium was used as the carrier gas with a linear velocity of 20 cm/s. The split ratio was 1:100, and the injection volume was 1 µL.

The α-tocopherol content was analyzed following the method described by Quispe-Fuentes et al. (2020) [27]. The procedure involved weighing 25 mg of extract, which was then mixed with 1 mL of methanol/BHT and stirred for 3 min. The resulting supernatant was filtered using 0.45 µm syringe filters and injected into an HPLC system. Quantification of α-tocopherol was conducted using HPLC with fluorescence detection. The analysis employed a Kromasil 100-5 C18 column (250 × 4.6 mm) with a mobile phase composed of methanol (1:1 *v*/*v*) at a flow rate of 1.2 mL/min. The fluorescence detector was set at excitation and emission wavelengths of 295 nm and 325 nm, respectively. Measurements were performed in triplicate, and the results were expressed as µg of α-tocopherol/g extract.

### 4.5. Ultrasound/Solvent Extraction

The extraction of bioactive compounds will be conducted using freeze-dried samples and an aqueous methanol solution, following the methodology described by Chandra Singh, Probst, Price, and Kelso (2022) [73], with modifications. The process was carried out using an ultrasonic bath (BIOBASE UC-30si, Jinan, China), operating at a frequency of 40 kHz. For each run, 1.0 g of berry powder was mixed with 12 mL of methanol-water solution at a 1:12 (*w*/*v*) ratio. The extraction was performed in a water bath maintained at 35 °C. Key parameters: extraction time (15, 25, and 35 min), ultrasound power (40, 60, and 80%), and solvent concentration (50, 70, and 90%), were optimized through a response surface methodology, as detailed in Section 4.6. After sonication, the mixture was filtered through Whatman No. 1 paper, and the solid residue was re-extracted under the same conditions. Combined extracts were stored at −80 °C until further analysis.

### 4.6. Optimization of Extraction Method

The experimental design consists of three factors with three levels each: time extraction (15, 25, and 35 min), ultrasound power (40, 60, and 80%), and solvent concentration (50, 70, and 90%), obtaining 27 combinations, respectively. These combinations were created using Rstudio software (version 2024.04.2) with the AlgDesign library and then optimized using the Box–Behnken model, reducing to 17 representative combinations, as shown in Table 3. The factors were codex by the coded.data function according with coded data = ((factor − center point)/amplitude). The experiments will be carried out in triplicate using freeze-drying Cauchao berry samples. The response surface methodology (RSM), implemented through the RSM library in RStudio, was utilized to determine the optimal combination of factors that maximize the extraction of bioactive compounds, evaluated by their antioxidant capacity using the DPPH assay.

### 4.7. Total Content of Phenolics, Flavonoids, and Anthocyanins

The total bio-compound content was evaluated in optimized Cauchao berry extract for total phenolic content (TPC), total flavonoid content (TFC), and total anthocyanin content (TAC).

The total phenolic content was determined using the Folin–Ciocalteu assay, following the method described by Rodríguez et al. (2014) [74] with slight modifications. Briefly, 15 µL of the extract was combined with 100 µL of Folin–Ciocalteu reagent (0.2 N) in a microplate. Subsequently, 100 µL of sodium carbonate solution (60 mg/mL) was added, and the mixture was incubated in the dark at room temperature for 90 min. After incubation, the TPC was quantified by measuring the absorbance at 750 nm using a multi-plate reader (Perkin-Elmer, Victor™ X3, Turku, Finland). A calibration curve was constructed using gallic acid as the standard, and TPC results were expressed as milligrams of gallic acid equivalents (GAE) per gram of dry matter (d.m.).

The total flavonoid content was quantified following the methodology outlined by Dini et al. (2010) [75] with minor adjustments. A volume of 500 µL of the optimized pomace extract was mixed with 150 µL of sodium nitrite (5%) and vortexed thoroughly. The mixture was left to rest for 5 min before adding 150 µL of aluminum chloride hexahydrate (AlCl_3_·6H_2_O, 10%). It was then incubated at room temperature in the dark for 6 min. To terminate the reaction, 1 mL of sodium hydroxide (NaOH, 1 M) was added, followed by the addition of 1.2 mL of ultrapure water. The absorbance of the final mixture was measured at 415 nm using a spectrophotometer (Thermo Scientific, ORION AQUAMATE 8000, Madison, WI, USA). A calibration curve was constructed using quercetin as the standard, and TFC values were expressed as milligrams of quercetin equivalents (QE) per gram of dry matter (d.m.).

The total anthocyanin content (TAC) was determined using the pH differential method, as described in De Souza et al. (2014) [76]. The red cabbage extract was diluted separately in buffers with pH 1.0 and pH 4.5. Absorbance measurements were taken at 510 nm and 700 nm for both buffer solutions. The difference in absorbance (*A*) was calculated using Equation (2):

(2) $$A=(A_{510}-A_{700}){pH}_{1.0}-(A_{510}-A_{700}){pH}_{4.5}$$

The total anthocyanin content, expressed as cyanidin-3-glucoside equivalents, was computed using the Equation (3):

(3) TAC=(A×MW×DF×Ve×1000)/(ε×1×M)

In the Equation (2), MW represents the molecular weight of cyanidin-3-glucoside (449 g/mol), DF denotes the dilution factor, Ve is the volume of the extract, ε corresponds to the molar extinction coefficient of cyanidin-3-glucoside (26,900 L·mol^−1^·cm^−1^), and M refers to the mass of the sample used for extraction. This method provides a reliable quantification of anthocyanins by leveraging their pH-dependent color changes, making it a widely used approach for evaluating anthocyanin content in plant-based extracts.

### 4.8. Antioxidant Potential

The functional properties were determined in optimized Cauchao berry extract. The antioxidant capacity was evaluated using the DPPH assay, following the method described by Grajeda-Iglesias et al. (2016) [77] with minor modifications. For the assay, 20 µL of red cabbage extract was mixed with 180 µL of freshly prepared DPPH solution (120 µM in 80% methanol) in 96-well microplates. The reaction mixture was incubated for 30 min in a multi-plate reader (Perkin-Elmer, Victor™ X3, Hamburg, Germany). Trolox served as the standard for constructing a calibration curve ranging from 1 to 500 µM (y = −0.001*x* + 0.5855; R^2^ = 0.9921). Absorbance was measured at 510 nm, and the results were expressed as µmol Trolox equivalents (TE) per gram of dry matter (d.m.).

The ORAC (Oxygen Radical Absorbance Capacity) assay was conducted according to the methodology described by Uribe et al. (2014) [78]. In a 96-well microplate reader (Perkin Elmer, Victor™ X3, Hamburg, Germany), 50 μL of red cabbage extract was mixed with 40 μL of phosphate buffer (pH 7.4). Subsequently, 200 μL of fluorescein (100 nM) was added to each well, and the mixture was incubated at 37 °C for 20 min. Following incubation, 35 μL of an AAPH solution (0.36 M) was added to initiate the reaction. Fluorescence readings were performed at excitation (λex) and emission (λem) wavelengths of 485 nm and 535 nm, respectively. The calibration curve for the ORAC assay was generated by plotting Trolox concentrations (5–250 μM) against the area under the fluorescence decay curve, yielding the equation: y = 0.00002*x* − 26.664 (R^2^ = 0.9769). Results were expressed as Trolox equivalents (µmol TE) per gram of dry matter (d.m.).

### 4.9. α-Glucosidase Activity

The enzymatic activity of α-glucosidase was assessed following the method described by Quispe-Fuentes et al. (2023) [79], with minor modifications. In a 96-well microplate, 50 µL of optimized Cauchao berry extract at varying concentrations (0.2–2 mg/mL) was mixed with 100 µL of α-glucosidase (0.5 U/mL; Saccharomyces cerevisiae, Sigma G5003, Ronkonkoma, NY, USA) and incubated at 25 °C for 10 min. After the preincubation period, 50 µL of 4-nitrophenyl α-D-glucopyranoside (Sigma N1377, St. Gallen, Switzerland) dissolved in 0.1 M phosphate buffer (pH 6.9) was added. The absorbance was measured at 405 nm using a Victor™ 3 multi-label plate reader, with the readings compared to a control containing 50 µL of buffer solution, representing the maximum enzymatic activity. Measurements were recorded every 30 s for 10 min. Sample and concentration were tested in triplicate, and α-glucosidase activity was calculated as a percentage based on the slope of the resulting exponential curve. Acarbose was used as a reference standard to compare the IC_50_ values of the extract.

### 4.10. Phenolic Compound Profiles

Three injections (20 µL) of each sample were made in the quantification of phenolic compounds was performed by HPLC-DAD analysis (Shimadzu Corporation, Kyoto, Japan) equipped with a Nucleosil^®^ 300 C18 column (25.0 cm × 4.6 mm; 5 µm particle size) [80]. The mobile phase comprised two solvents: eluent A was a mixture of water and formic acid (99:1, *v*/*v*), and eluent B was acetonitrile. The solvent system started with 8% of B, rising to 15% after 25 min, 22% after 55 min, and 40% after 60 min. The calibration curves were obtained from standard solutions at six different concentrations; the graphs of these showed a good correlation of 0.99 for all standards (cyanidin 3-O-glucoside derivative 1, cyanidin 3-O-glucoside derivative 2, quantified as petunidin 3-O-ruthinoside, and quercetin derivatives were quantified as quercetin 3-O-glucoside).

## 5. Conclusions

This study identifies Cauchao berry as a novel and promising native source of bioactive compounds with potential antioxidant and antidiabetic properties. The optimized extraction process yielded a phytochemical-rich profile characterized by high levels of total phenolics, flavonoids, anthocyanins, essential fatty acids, and α-tocopherol. These compounds could be responsible for the observed antioxidant activity and α-glucosidase inhibition. Furthermore, the presence of chlorogenic and sinapic acids suggests a potential role in mitigating oxidative stress, metabolic dysfunction, and neurodegenerative conditions, as supported by previous literature. This study provides a pioneering contribution to the scientific characterization of Cauchao berries and highlights their potential as a valuable native resource for the development of functional foods and nutraceuticals derived from underexplored species. Future studies should be directed toward exploring the neuroprotective effects of these compounds, particularly in models of oxidative damage and neurodegeneration.

## Figures and Tables

**Table 1:** Proximate composition and dietary fiber content of Cauchao berries.

Parameters (g/100 g)	Fresh	Freeze-Dried
Moisture	75.31 ^a^ ± 0.41	2.78 ^b^ ± 0.10
Fat	2.15 ^b^ ± 0.15	9.15 ^a^ ± 0.16
Ash	0.57 ^b^ ± 0.03	2.50 ^a^ ± 0.15
Crude protein	1.73 ^b^ ± 0.17	7.15 ^a^ ± 0.23
Crude fiber	3.94 ^b^ ± 0.67	15.25 ^a^ ± 0.50
Carbohydrates	20.24 ^b^ ± 0.32	78.42 ^a^ ± 0.29
Reducing sugars	7.71 ^b^ ± 0.42	39.82 ^a^ ± 1.58
Insoluble dietary fiber *	34.78 ^a^ ± 1.73	34.26 ^a^ ± 2.55
Soluble dietary fiber *	4.60 ^a^ ± 0.50	3.66 ^a^ ± 0.81
Total dietary fiber *	39.37 ^a^ ± 2.23	37.92 ^a^ ± 3.36

Values are expressed as mean ± standard deviation (*n* = 3). * Dry matter. Different letters in the same row indicate significant differences between treatments (*p* < 0.05), according to the Student’s *t*-test.

**Table 2:** Fatty acids and α-tocopherol content in Cauchao berries.

Fatty Acids (g/100 g Fatty Acid)	Fresh	Freeze-Dried
C16:0	6.99 ^a^ ± 0.02	7.18 ^a^ ± 0.13
C18:0	2.62 ^b^ ± 0.04	2.71 ^a^ ± 0.02
C18:1n9c	8.83 ^b^ ± 0.05	9.24 ^a^ ± 0.17
C18:2n6c	80.29 ^a^ ± 0.06	79.44 ^b^ ± 0.03
C20:0	0.35 ^a^ ± 0.07	0.41 ^a^ ± 0.01
C20:1	0.92 ^a^ ± 0.06	1.02 ^a^ ± 0.04
α-tocopherol (µg/g)	95.51 ^a^ ± 5.42	105.41 ^a^ ± 1.39

Values are expressed as mean ± standard deviation (*n* = 3). Different letters in the same row indicate significant differences between treatments (*p* < 0.05). Statistical analysis was performed using the Student’s *t*-test for all variables except C20:0, which was analyzed using the Mann–Whitney test due to non-normal distribution.

**Table 3:** Box–Behnken model, coded factor, and DPPH results.

Factors			Coded Factor			Result
Solvent (%)	Power (%)	Time (min)	X_1_	X_2_	X_3_	DPPH (μmol TE/g d.m)
90	80	25	0	1	1	185.89
50	60	35	1	0	−1	248.70
70	40	35	1	−1	0	246.87
50	40	25	0	−1	−1	254.67
90	60	35	1	0	1	170.16
90	60	15	−1	0	1	169.52
70	60	25	0	0	0	261.32
90	40	25	0	−1	1	191.53
70	60	25	0	0	0	237.68
50	60	15	−1	0	−1	205.62
50	80	25	0	1	−1	277.65
70	60	25	0	0	0	243.76
70	60	25	0	0	0	218.70
70	80	15	−1	1	0	222.84
70	40	15	0	−1	0	254.89
70	80	35	1	1	0	246.09
70	60	25	0	0	0	251.31

**Table 4:** Bioactive compound content and antioxidant potential of optimized Cauchao berry extract.

Parameter	Value (Mean ± SD)
TPC (mg GAE/g d.m.)	25.43 ± 0.85
TFC (mg QE/g d.m.)	46.51 ± 1.38
TAC (mg cyanidin-3-glucoside/g d.m.)	5.91 ± 0.40
DPPH (μmol TE/g d.m.)	289.54 ± 9.05
ORAC (μmol TE/g d.m.)	451.09 ± 6.04

Values are expressed as mean ± standard deviation (*n* = 3).

## Data Availability

Data is contained within the article.
